# Editorial

**Published:** 1850-10

**Authors:** 


### AMERICAN SOCIETY OF DENTAL SURGEONS.

The reported discussions of the American Society of Dental Surgeons at their ninth annual meeting held at Saratoga, August, 1848, possess more than ordinary interest. We have here a Synopsis, as it were, of each member's practice, and that, too, on a very important subject—the separating and plugging of teeth. As we were not permitted to participate in these discussions orally, we hope in our next o add our mite.

The object of such meetings is to compare practice, and thus elicit useful knowledge. Judging from the report, so far published; we should think the meeting one of great interest.

---

### DENTAL REMOVAL.

Dr. J. S. King of Lancaster Ohio, has located in Pittsburgh. Dr. King was a student in our office near three years, attended two courses in the Dental College in this City; and graduated as we think with honor to himself and Alma Mater. Such a preparatory course should insure success. And we can with pleasure commend the Dr. to the profession of our sister city, and also, to the public, as one every way worthy of their confidence.

---

In the last No. of the Register we noticed the fourth edition of Dr. Harris's work, on the principles and practice of dental Surgery. We should then have added that it is for sale by Dr. Brown at Dental Depot, corner of 4th., and Walnut Sts.

---

The Committee selected to examine the graduating class of the Ohio College of Dental Surgery, at its next commencement, is composed of the following gentlemen.

*From the Medical Profession.*

Charles Woodward, M. D.
Israel Dodge, M. D.

*From the Dental Profession.*

Dr. Clute, Louisville, Ky.
Dr. W. E. Ide, Columbus, Ohio.
Dr. J. Allen, Cincinnati.

### BACK NOS. OF THE REGISTER.

The first, second and third volumes will be furnished to new subscribers (who will pay in advance for the fourth volume,) at the reduced price of five dollars.

---

The present No. of the Register will be sent to many who we think, should sustain such a work, and if not returned we shall consider them as subscribers, and regularly mail it to them.

---

### TO DELINQUENT SUBSCRIBERS.

We hope that such will remember, that the printer must be paid; and that the Register does not afford a salary to the editor.

---

We see by the Dental News letter, that Drs. L. Roper of Philadelphia, and C. Blakeley of Havana Cuba, are both added to the list of the departed, Dr. Roper on his return from California.

It is also supposed that Dr. L. Koecker, of London is dead.

---

### OBITUARY.

We regret to announce the decease of Dr. John Jones, of Dayton, Ohio.

Dr. Jones had been engaged in the practice of the profession, about twenty years—was a student of my brother now in business with me. Shortly after commencing practice, he paid a visit to the Miamia valley, and soon took up his permanent abode in it; practicing for several years at Miamisburg and neighboring towns, but ultimately removed to Dayton, where he spent the last eight or ten years—Dr. Jones maintained a high standing in his profession, and those who knew him well, could only justly appreciate his private worth; a friend in Dayton writing of his death, thus remarks: "a man highly esteemed both in professional and private life; his loss is no doubt felt by more of the citizens than would be, the loss any other citizen, either public or private."

He deceased on the eleventh of September,—aged near 50.

MEDICAL COLLEGE OF OHIO.—We are glad to see an unusual number of students in attendance on the preparatory lectures of this Institution. The faculty are all now at their posts and judging from the general expression of opinion among our medical men, the pros pects of this school were never better.

The Gentlemen who have been recently added to the faculty bring with them, character, reputation and talent.

---

### Medical College of Ohio.

### Session of 1850-'51.

THE THIRTY FIRST ANNUAL SESSION OF THIS INSTITUTION, will open on the FIRST MONDAY IN NOVEMBER NEXT, and close on the last of February, under the following arrangements:

H. WILLIS BAXLEY, M. D., Professor of Anatomy.

JOHN LOCKE, M. D., Professor of Chemistry and Pharmacy.

L. M. LAWSON, M. D., Professor of Physiology and Phathology.

T. O. EDWARDS, M. D., Professor of Materia Medica and Therapeutics, and Medical Jurisprudence.

R. D. MUSSEY, M. D., Professor of Surgery.

LANDON C. RIVES, M. D., Professor of Obstetrics and the Diseases of Women and Children.

JOHN BELL, M. D., Professor of Theory and Practice of Medicine.

JOHN DAVIS, M. D., Demonstrator of Anatomy.

☞ The following branches will be included in the course: *Anatomy, Chemistry, Pharmacy, Physiology, Pathology, Materia Medica, Therapeutics, Medical Jurisprudence, Medical Botany, Surgery, Obstetrics, Diseases of Females, Diseases of Children, Practical Medicine,* and *Physical Diagnosis.*

☞ The DISSECTING ROOMS will be opened for classes on the 1st of October.

☞ CLINICAL LECTURES, on Medicine and Surgery, will be delivered at the Commercial Hospital three times a week.

### October Lectures.

A Course of Lectures will be delivered by the Faculty, (free of charge,) commencing on the first of October, and embracing the following subjects:

*Anatomy and Physiology of the Senses; Diseases of the Eye; Medical and Elementary Botany; Functional and Organic Diseases of the Uterus; Medical Jurisprudenee; Physical Diagnosis.*

☞ Also, CLINICAL LECTURES at the Commercial Hospital.

FEES.—For a full Course of Lectures, $84; Matriculation and Library Ticket, $5; Dissecting Ticket, $8; GraduationFee, $20; HospitalTicket, $5.

☞ Board (including the expenses of room, fuel, and lights,) can be obtained at from $2 to $3 per week.

Further information may be obtained by addressing the Dean.

L. M. LAWSON, M. D., *Dean of the Faculty.*
South side of Sixth st., between Walnut and Vine.

Cincinnati, Nov., 1850.

